# 

**Published:** 1867-04

**Authors:** 


### THE CHICAGO MEDICAL EXAMINER

A MONTHLY JOURNAL, DEVOTED TO THE

EDUCATIONAL, SCIENTIFIC, AND PRACTICAL INTERESTS

OF THE

MEDICAL PROFESSION.

EDITED BY

N. S. DAVIS, M.D.,

PROFESSOR OF PRINCIPLES AND PRACTICE OF MEDICINE AND OF CLINICAL MEDICINE, ETC., ETC.

TERMS, $3.00 PER ANNUM, IN ADVANCE.

CHICAGO:
GEORGE H. FERGUS, BOOK AND JOB PRINTER,
12 CLARK STREET.

